# Supplementary material for: Hepatitis E Virus in People Who Use Crack-Cocaine: A Cross-Sectional Study in a Remote Region of Northern Brazil
Source: Viruses. 2021 May 17;13(5):926. doi: 10.3390/v13050926 (PMC8156048; doi:10.3390/v13050926)
Supplement: Supplementary file 1 [file viruses-13-00926-s001.zip › viruses-1166321-supplementary.pdf]

Description of extra procedure used in this study.

Isolation of viral RNA from stool using the QIAamp® Viral RNA Mini Kit.

Please be sure to read the QIAamp® Viral RNA Mini Kit Handbook (Qiagen) carefully before beginning this procedure.

Step by step:

1. Suspend 0.5 - 1.0 ml stool in up to 5 ml of 0.89% NaCl (i.e., up to 1:10 dilution).
2. Clarify the solution by centrifugation for 20 min at 4000 x g.
3. Filter the supernatant using a 0.22 μm filter.
4. Use 140 μl of the filtrate as starting material following the Viral RNA Mini Spin Protocol or the Viral RNA Mini Vacuum Protocol in the QIAamp Viral RNA Mini Kit Handbook.

For more details, visit: https://www.qiagen.com/us/resources/resourcedetail?id=cee75d34-6d1f-4f44-9039-9597b708e6a2&lang=en

Table S1. Nucleotide sequences (from the 5′ terminal region of ORF1 (255 nt)) of hepatitis E virus and other information used to reconstruct the maximum likelihood phylogenetic tree presented in this study.

Genotype/subtype	GenBank access number (HEV host)	Evolutionary model
1	LC061267 (Dromedary camel), NC_001434 (Monkey)	GTR + G *
2a	M74506 (Man)	
3a	AB089824 (Man), AB074920 (Man), AB082552 (Man), AF060668 (Swine)	
3b	AB189072 (Man), AB443626 (Swine), FJ527832 (Swine), AB189071 (Deer), AB091394 (Man)	
3c	EF591852 (Swine), EF591853 (Swine), HM154537 Swine), JN983199 (Man), JN983203 (Man)	
3e	AJ315768 (Man), AF110389 (Man), FJ998015 (Boar)	
3f	AB369687 (Man), AF336005 (Swine)	
4	AB220977 (Man), AB220978 (Man), AB220979 (Man)	

* Akaike information criteria.

Table S2. Factors not associated with hepatitis E virus exposure in people who use crack-cocaine in a remote region of northern Brazil.

Factors	Total	HEV + (%)	Bivariate OR (95% CI)	Multivariate OR (95% CI)
Male *versus* (*vs.*) Female	314	59 (18.8)	0.8 (0.4 - 1.5)	1.0 (0.6 - 2.0)
Age ≥ 30 years *vs.* > 30 years	146	24 (16.4)	0.8 (0.5 - 1.4)	1.2 (0.7 - 1.9)
Non-white *vs.* White	389	76 (19.5)	3.0 (0.9 - 10.2)	3.7 (0.8 - 9.8)
Single, separated or widowed *vs.* Married or cohabitating†	407	77 (18.9)	3.2 (0.7 - 13.0)	3.4 (0.6 - 13.7)
Up to elementary school *vs.* High school or more	353	67 (19.0)	1.4 (0.7 - 2.9)	2.1 (0.5 - 3.3)
Heterosexual *vs.* Same sex (including bisexual)	396	75 (18.9)	2.0 (0.7 - 6.1)	1.8 (0.6 - 4.5)
Unprotected sex *vs.* Protected sex†	396	76 (19.2)	3.0 (0.8 - 9.3)	3.5 (0.7 - 10.1)
More than 10 sexual partners *vs.* Up to 10 sexual partners†	189	31 (16.4)	0.8 (0.4 - 1.4)	1.1 (0.5 - 1.7)
Oral sex *vs.* No oral sex†	281	47 (16.7)	0.7 (0.4 - 1.3)	0.8 (0.5 - 1.8)
Anal sex *vs.* No anal sex†	157	32 (20.4)	1.2 (0.7 - 2.1)	1.5 (0.8 - 3.0)
Exchange of sex for money/illicit drug *vs.* No exchange of sex for money/illicit drugs†	171	58 (33.9)	1.1 (0.6 - 2.0)	1.4 (0.5 - 2.4)

†Last 12 months. OR: Odds Ratio. 95% CI: 95% confidence interval. OR: Odds Ratio.
